# Assessing Health Care Providers’ Knowledge of Arthritis-Appropriate, Evidence-Based Interventions Through Online Self-Assessment

**DOI:** 10.5888/pcd23.250313

**Published:** 2026-03-19

**Authors:** Elizabeth A. Joy, Lindsey Gardner, Katie Lucero, Sara Thorpe, Meng Yuan, Elizabeth Erck, Heather Murphy, Shalu Garcha, Adam Burch, Anika L. Foster

**Affiliations:** 1 University of Utah, Exercise Is Medicine, Salt Lake City, Utah; 2 Medscape Education, Newark, New Jersey; 3 National Association of Chronic Disease Directors, Decatur, Georgia; 4 State of New Hampshire Department of Health, Derry, New Hampshire; 5 Unaffiliated, Atlanta, Georgia

## Abstract

**Introduction:**

Physical activity is an important nonpharmacological strategy for managing arthritis-attributable pain and improving physical function. To prepare health care providers (HCPs) to deliver this care, the National Association of Chronic Disease Directors, the Centers for Disease Control and Prevention, and Medscape Education developed an online clinical practice assessment activity (CPA). This activity aimed to help HCPs assess their knowledge of arthritis-appropriate, evidence-based interventions (AAEBIs) and better incorporate them into treatment programs.

**Methods:**

A 27-question CPA was launched in November 2022 and analyzed in May 2025. Educational effectiveness was assessed with a repeated-pairs preassessment/postassessment. Responses to multiple choice and confidence questions were aggregated and analyzed to help us understand participants’ current practices and education needs for the management of patients with arthritis.

**Results:**

Approximately 20,000 HCPs accessed the CPA; 4,395 completed the assessment and were included in the analysis. Less than two-thirds (59.8%) correctly identified recommendations about weekly moderate-intensity physical activity for adults with arthritis. After participating in the CPA, 82.2% agreed or strongly agreed that their awareness of AAEBIs and their benefits had increased.

**Conclusions:**

HCPs showed a need for education to reinforce, improve, or extend knowledge about arthritis risk factors, barriers to physical activity, and physical activity prescription.

**Summary**

**What is already known about this topic?**

Health care delivered by primary and specialty providers can assist arthritis patients in achieving recommended levels of physical activity. Yet, providers often fail to discuss physical activity with patients regarding arthritis management.

**What is added by this report?**

Notable knowledge gaps exist for health care providers related to physical activity interventions that can reduce pain and improve function for patients with arthritis.

**What are the implications for public health practice?**

Improving awareness among health care providers about arthritis and the effect of regular physical activity on pain, mobility, and function, and improving awareness of arthritis-appropriate evidence-based interventions can improve patient outcomes and enhance quality of life.

## Introduction

During 2019–2021, an estimated 53.2 million adults in the US were diagnosed with arthritis (1); that number is expected to rise to 78.4 million by 2040 (2). Arthritis is associated with joint pain, swelling, decreased joint mobility, and reduced functional range of motion, which can lead to increased sedentary behavior and a decline in regular physical activity (3–5). As a result, over 40% of adults with arthritis report limitations in their activities due to arthritis (6). Additionally, people with arthritis face higher risk of falls and fractures (7) and incur more than $2,000 in extra annual health care costs (8).

Physical activity is a nonpharmacological strategy for managing arthritis-attributable pain and improving physical function (9,10). Physical activity is defined as any sustained body movement that increases energy expenditure; exercise is a subcategory of physical activity that is planned, purposeful, and repeated on a regular basis to improve or maintain health and fitness (11). The *Physical Activity Guidelines for Americans, 2nd edition*, recommends that all older adults (aged ≥65 years), including those with any form of arthritis, engage in at least 150 minutes per week of moderate-intensity (or 75 minutes of vigorous-intensity; or an equivalent combination of moderate- to vigorous-intensity) aerobic exercise, and 2 days per week of muscle-strengthening exercise (9). Guidelines from the Centers for Disease Control and Prevention (CDC) and American College of Rheumatology emphasize both physical activity and exercise: encouraging people with arthritis to stay physically active overall while also performing exercises to strengthen muscles around the joints, maintain range of motion, and support long-term joint health (12,13). Engaging in this level of physical activity has been shown to reduce pain, improve mobility, and increase function and quality of life for people affected by arthritis (9,14,15). In addition to regular physical activity and exercise, people with arthritis should be encouraged to seek movement throughout the day and reduce sedentary time (16).

Arthritis-appropriate, evidence-based interventions (AAEBIs) are a collection of community-based programs validated as safe and effective for increasing physical activity levels among people with arthritis (15,17–20). Despite the known benefits of regular physical activity, only 24.2% of US adults meet the guidelines for both aerobic and muscle-strengthening activities; this number drops to only 15% for adults aged 65 or older (21). These statistics are especially relevant considering that the prevalence of arthritis increases with age, from 11.7% in adults aged 18 to 44 years to 48.3% for those aged 65 years or older (1).

People with arthritis may seek care from various health care providers (HCPs) including primary and specialty care physicians, advanced practice providers (eg, nurse practitioners, physician assistants), nurses, and allied health professionals — all of whom have differing educational backgrounds and awareness regarding AAEBIs. HCP barriers to making referrals for exercise include lack of time, lack of feedback regarding the patients referred, medico-legal responsibility, a feeling that patients may not take exercise advice given, and the belief that physical activity promotion is not a priority during routine consultations (22,23). To address variations in HCP knowledge and attitudes toward referring people with arthritis to AAEBIs, a web-based clinical practice assessment (CPA), *Lifestyle Management Programs for Arthritis: Expand Your Knowledge on Evidence-Based Interventions* (www.medscape.org/viewarticle/983780), was developed and promoted to increase HCP referrals to AAEBIs and to establish a baseline of their understanding of AAEBIs, current practices, and educational gaps.

## Methods

The National Association of Chronic Disease Directors, CDC, Medscape Education, and E.A.J. (representing Exercise Is Medicine), developed a 27-question online CPA. Design of the CPA was a joint effort involving multiple subject matter experts from the partnering organizations, ensuring that the CPA reflected both practical field experience and best practices in arthritis management. CPAs are questionnaire-style continuing medical education or continuing education activities (“self-assessment survey”) that assess clinicians’ knowledge and practice around a particular topic. The online activity page presents goals and objectives, and learners then complete multiple-choice test items and an evaluation to earn credit. The CPA also delivers education following responses on why the right answer was the best answer. Paired preassessment and postassessment CPA activity questions compare responses before and after educational content is presented to gauge change in knowledge and competence. The CPA is posted on Medscape Education and jointly promoted by Medscape and partnering organizations to people in relevant specialties for participation and credit. Results are summarized by using Medscape’s outcomes framework (eg, Moore’s levels of CME outcomes) and compiled into metrics and outcomes reports.

The CPA covers various arthritis topics including background, the public health threat of arthritis, osteoarthritis pathophysiology, benefits of physical activity, awareness and use of screening tools, current practices in physical activity counseling, and referrals to AAEBIs (Table 1). The question and answer-based learning design provided an opportunity to establish a baseline of HCPs’ knowledge, skills, attitudes, and competence regarding the management of patients with arthritis. Immediate feedback was used as the educational component to allow learners to receive instant information regarding their performance, understanding, or progress. This approach not only reinforced comprehension for those who answered correctly but also enhanced knowledge, competence, and confidence for those who answered incorrectly. The CPA also includes a resource library that offers supplementary tools and resources for HCPs to integrate into their clinical workflow when caring for people with arthritis.

**Table 1:** Clinical Practice Assessment Questions About Lifestyle Management Programs for Arthritis, by Topic Area and Representative Questions, November 28, 2022, Through December 31, 2024

Topic area/representative question	Number of CPA questions
Confidence questions preactivity Example question: How confident are you right now in your ability to conduct physical activity screenings to assess the need for arthritis interventions, and to counsel patients about activity and exercise to ease arthritis pain?	2
Background knowledge Example question: What is the most common clinician-reported barrier to treating patients with arthritis-appropriate, evidence-based interventions (AAEBIs)?	3
Public health threat of arthritis Example question: The currently available literature suggests that which of the following racial/ethnic populations has the highest incidence rate of OA pain in the United States?	4
Osteoarthritis pathophysiology Example question: You are seeing a male patient who broke his ankle in a football accident 10 years ago. He is a smoker and has a preexisting autoimmune disorder. What is considered a risk factor for developing OA?	1
Benefits of physical activity Example question: What is the current CDC recommendation regarding weekly moderate-intensity physical activity for adults with arthritis?	3
Role of health care providers in encouraging self-management and physical activity Example question: What treatment strategy is currently strongly recommended by the 2019 American College of Rheumatology (ACR)/Arthritis Foundation guidelines for the management of OA of the hip and/or knee for patients who are overweight or obese?	1
Screening tools Example question: The American College of Sports Medicine endorses which tool to quantify a patient’s amount of exercise?	2
Counseling about the benefits of physical activity and role of self-management Example question: A patient with a BMI of 35 presents with chronic knee and hip pain. Imaging shows evidence of OA in the hip and both knees. Which of the following interventions is unlikely to improve the patient’s pain in the long term?	4
Referral to AAEBIs and other treatment options Example question: According to the Arthritis Foundation, which activity is the least likely to worsen pain in patients with arthritis?	2
Confidence questions postactivity Example question: How confident are you right now in your ability to prescribe arthritis-appropriate evidence-based interventions to your patients?	2
Learning segmentation questions Example question: Which of the following most closely describes your specialty?	3
**Total Questions**	**27**

Abbreviations: BMI, body mass index; CDC, Centers for Disease Control and Prevention; OA, osteoarthritis.

The structure and components of the CPA are outlined below, illustrating how the activity enables back-end analysis to measure changes in learner confidence, gather data for segmentation, and solicit feedback on the educational experience.

- The CPA process begins with 2 baseline Likert scale confidence questions evaluating how prepared learners feel to care for patients with arthritis.
- Next, learners proceed through 20 educational, multiple-choice questions on arthritis care.
- The same 2 confidence questions are presented again at the end to measure changes in confidence.
- After the postconfidence questions, learners complete 3 wrap-up questions, allowing for segmentation of the data by learner characteristics, focused on increased awareness of AAEBIs, the learner’s specialty area, and patient referral mechanisms.
- After completing the CPA, learners are offered an optional feedback questionnaire with various formats (multiple-choice, Likert, checkbox, open-ended) to gather learner satisfaction and intended changes to clinical practice.

Medscape is the leading resource for clinical and medical scientists seeking access to medical information (24). The CPA became available to Medscape members in November 2022. Learners had the opportunity to earn up to 0.25 American Medical Association Physician’s Recognition Award Category 1 Credit(s) or up to 0.25 contact hour(s) of nursing continuing professional development credit through December 30, 2025.

For this analysis, we included only learners who 1) completed the activity in full, defined as answering questions 1 through 25, and 2) self-identified in response to question 26 as internal medicine or primary care physician, nurse practitioner, physician assistant, nurse, geriatrician, orthopedic surgeon, rheumatologist, sports medicine specialist, physical therapist, chiropractor, pharmacist, acupuncturist, occupational therapist, during November 28, 2022, through December 31, 2024. These specialties represent the core members of the multidisciplinary team commonly involved in the assessment and management of osteoarthritis and chronic musculoskeletal pain (25).

Educational effectiveness was assessed by using 2 repeated Likert scale questions at the beginning and end of the CPA to measure changes in learners’ confidence resulting from their participation. Additionally, satisfaction indicators gathered through an optional postactivity evaluation survey provided information on educational impact in terms of intent to change practices. Gaps in knowledge and competence were measured for each CPA educational content question by the percentage of learners who selected incorrect answers, whereas knowledge was measured by the percentage of learners who selected the correct answer. To determine whether the CPA influenced learners’ confidence, we conducted a repeated-measures *t* test with Cohen’s d used to measure effect size. We used Python 3.0 (Python Software Foundation) to execute paired sample *t* tests and calculate Cohen’s d for each confidence question. Knowledge and competence data were analyzed and presented as aggregate absolute values, indicating the number and percentage of learners who answered a question correctly. Tableau (Salesforce Inc) was used to conduct accurate calculation and analysis of percentages.

## Results

### Participation

Within the broader activity reach, the online self-assessment page received 20,023 unique page views on the first page of the activity, meaning it was accessed in 20,023 individual HCP sessions. A total of 4,395 HCPs met the inclusion criteria for this analysis, having completed all required assessment questions and self-identified within 1 of 13 clinical specialties. HCPs were categorized into 3 groups based on health care specialty: primary care, specialist, and therapeutic care (Figure, Table 2).

**Figure:**
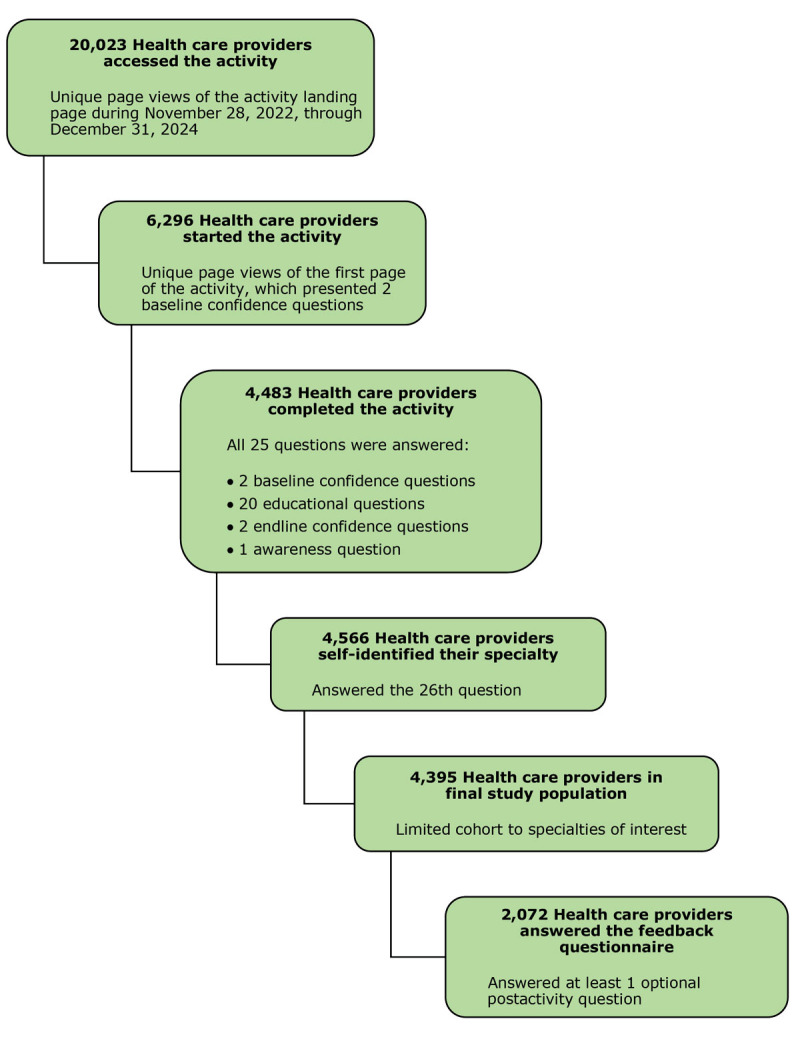
Defining the study population through stepwise eligibility criteria for a clinical practice assessment about lifestyle management programs for arthritis, November 28, 2022, through December 31, 2024.

**Table 2:** Percentage of Incorrect Responses to Knowledge and Clinical Decision-Making Questions, Stratified by Health Care Provider Specialty, for a Clinical Practice Assessment About Lifestyle Management Programs for Arthritis, November 28, 2022, through December 31, 2024

Specialty	N (%)	Prevalence of arthritis and arthritis- associated activity limitations, %	Physical activity recommendations for adults with arthritis, %	Physical activity to reduce pain and improve function, %	Evidence-based assessment steps for evaluating physical activity, %	Comorbidities associated with the development of osteoarthritis, %
**Primary**	**3,018 (68.7)**	**72.4**	**57.2**	**60.6**	**72.3**	**95.0**
Primary care physicians or internal medicine	1,190 (27.1)	69.2	58.0	65.5	76.9	94.0
Geriatric medicine	150 (3.4)	72.0	68.0	73.3	77.3	82.7
Nurses	1,091 (24.8)	74.5	57.2	54.2	66.8	96.9
Nurse practitioners	319 (7.3)	73.7	48.3	55.2	68.7	97.2
Physician assistants	268 (6.1)	76.5	58.2	64.6	75.4	95.5
**Specialty**	**1,012 (23.0)**	**74.0**	**65.2**	**63.9**	**73.5**	**93.7**
Orthopedic surgeons	640 (14.6)	74.8	69.5	63.6	73.1	94.2
Rheumatologists	159 (3.6)	70.4	70.4	72.3	74.2	88.7
Sports medicine	213 (4.8)	74.2	48.4	58.7	74.2	95.8
**Therapeutic**	**365 (8.3)**	**75.6**	**66.8**	**60.5**	**69.0**	**90.1**
Acupuncturists	16 (0.4)	81.3^a^	75.0	81.3^a^	68.8	75.0^a^
Chiropractors	63 (1.4)	69.8	63.5	57.1	68.3	90.5
Occupational therapists	42 (1.0)	71.4	66.7	64.3	76.2	83.3
Pharmacists	81 (1.8)	74.1	71.6	60.5	71.6	92.6
Physical therapists	163 (3.7)	79.1	65.0	58.9	66.3	92.0
**Total**	**4,395 (100)**	**73.0**	**59.8**	**61.4**	**72.3**	**94.3**

a Denotes outlier.

Most HCPs (68.7%, n = 3,018) came from primary care specialties. The 3 most represented specialties among activity completers were internal medicine or primary care physicians (27.1%), nurses (24.8%), and orthopedic surgeons (14.6%). A smaller proportion of HCPs represented therapeutic care disciplines such as physical therapy (3.7%) and pharmacy (1.8%). In the optional postactivity feedback questionnaire, nearly three-quarters (72.2%; n = 1,469) of 2,034 HCPs report seeing at least 1 patient a month who may benefit from the content of the CPA.

### Satisfaction and impact

The postactivity evaluation questionnaire included optional questions to assess satisfaction. Most HCPs (98.7%, n = 1,890 of 1,915) agreed that the educational activity was presented objectively and was free of commercial bias. Also, 91.8% (n = 1,861 of 2,028) agreed that the CPA subject matter expert proved to be knowledgeable and effective and 87.4% (n = 1,727 of 1,977) indicated that they would recommend the CPA to others. Fewer HCPs agreed that their awareness had improved (62.6%) or committed to making 1 or more changes (76.1%).

In terms of CPA impact, 76.1% (n = 1,491) of HCPs reported being committed or very committed to changing their practices based on what they learned in the CPA. The top 5 practices HCPs intended to change, as identified through a “select all that apply” checkbox question, were modifying treatment plans (42.3%, n = 840), changing screening practices (16.9%, n = 335), collaborating with the health care team differently (16.4%, n = 326), communicating with patients, families, or caregivers differently (15.1%, n = 300), and incorporating different diagnostic strategies into patient evaluations (11.5%, n = 228). More than one-third of HCPs did not plan changes; of these, 34.1% (191 of 560) said the CPA validated their current approach, and for the remainder, our instrument did not ascertain reasons.

### Knowledge and competence gaps

Persistent gaps were identified related to the prevalence of arthritis, associated risk factors, physical activity guidelines, and tools for assessing physical activity patterns. For instance, 73.0% of HCPs were unaware of the prevalence of arthritis and arthritis-associated activity limitations. This lack of awareness was consistent across the 3 specialty groups, indicating a broad cross-disciplinary knowledge gap. Among the different specialties, 81.3% of acupuncturists, 79.1% of physical therapists, and 76.5% of physician assistants were unaware of the burden of arthritis and activity limitations among adults in the US.

More than half (59.8%) of HCPs did not know the recommendation that adults with arthritis should engage in at least 150 minutes per week of moderate physical activity and 2 days per week of strength training (9). Primary care specialists showed greater knowledge of the CDC recommendations for physical activity for adults, with nurse practitioners (48.3%) demonstrating the smallest knowledge gap. Despite multiple studies that physical activity can reduce pain and improve function in patients with arthritis, the scale of this benefit was incorrectly identified by nearly two-thirds (61.4%) of HCPs.

Many HCPs (72.3%) lacked knowledge regarding the full process of integrating evidence-based assessment steps for evaluating physical activity, as outlined by the *Exercise Is Medicine* global health initiative (26). Gaps in knowledge were highest among geriatricians (77.3%), primary care physicians (76.9%), occupational therapists (76.2%), physician assistants (75.4%), and rheumatologists and sports medicine providers (74.2%).

Most HCPs (94.3%) had difficulty applying knowledge about comorbidities linked to osteoarthritis in a question based on a case example. While there were slight differences between professional groups, overall baseline knowledge was low, with 90.1% answering incorrectly. HCPs in a few specialties showed stronger understanding, but gaps were consistent across most groups. The 3 specialties with the least knowledge regarding arthritis comorbidities were nurse practitioners at 97.2%, nurses at 96.9%, and sports medicine at 95.8%. Acupuncturists (75.0%), geriatric medicine (82.7%), and occupational therapists (83.3%) had the highest baseline knowledge.

Despite low baseline knowledge, after participating in the CPA, 82.2% of HCPs agreed or strongly agreed that their awareness of AAEBIs and their benefits had improved. Nurse practitioners (87.1%) had the highest level of agreement while acupuncturists (62.5%) had the lowest.

### Confidence and Collaboration

HCPs demonstrated a significant (*P* < .001) increase in both their confidence to prescribe AAEBIs from preeducation (mean [SD], 2.43 [1.14]) to posteducation (mean [SD], 2.96 [1.04]), with a medium effect size (d = −0.55), and their confidence to assess physical activity patterns from preeducation (mean [SD], 2.51 [1.12]) to posteducation (mean [SD], 2.93 [1.01]), with a small effect size (d = −0.44). Additionally, among HCPs, 92.1% (2,184 of 2,371) indicated that the activity would help them collaborate more effectively with other care-team members.

## Discussion

An online CPA was established for HCPs to 1) self-assess learning needs related to nonpharmacologic interventions for patients with arthritis; 2) be able to incorporate AAEBIs into patients’ self-management efforts; and 3) acquire and retain knowledge that will enhance their clinical practice and improve patient outcomes. The interest in the CPA from more than 20,000 HCPs underscores a potential demand for arthritis-related content among HCPs. The CPA attracted participation from various types of HCPs, with physicians accounting for 53.5% and other HCPs comprising the remaining 46.5%. This diverse representation included nurses, nurse practitioners, physician assistants, and pharmacists, reflecting the multidisciplinary approach to care for people with arthritis.

On completion of the CPA, HCPs were expected to demonstrate enhanced knowledge regarding AAEBIs and other relevant physical activity opportunities for patients with arthritis. Additionally, HCPs should be able to self-assess their learning needs regarding the benefits of physical activity in managing arthritis symptoms. Almost all HCPs reported that the activity would help them collaborate more effectively with other care-team members, underscoring the CPA’s relevance to interprofessional practice. In practical terms, such collaboration would reasonably include shared referral pathways to evidence-based physical activity or self-management programs, warm handoffs to other health care professionals, brief case conferencing, and use of electronic health record order sets that cue team-based care. Many participants reported an intention to change aspects of their clinical practice based on the CPA, suggesting movement beyond knowledge acquisition toward behavior change. Among those who did not anticipate changes, a notable subset indicated that the CPA validated their current approach. Given common implementation barriers in US settings (eg, time constraints, local policies or procedures, reimbursement, limited access to community programs), lack of planned change may reflect contextual constraints rather than the educational content. Taken together, these findings support the CPA as an educational catalyst while highlighting the need for accompanying system supports — clinic protocols, electronic health record prompts, and streamlined referral workflows — to translate learning into sustained interprofessional practice.

The findings of the CPA are relevant within the context of arthritis care. Knowledge gaps in several areas were identified among HCPs across all specialties. For example, 59.8% of HCPs did not know the current Physical Activity Guidelines for Americans recommendation (9) for adults with arthritis to obtain 150 minutes per week of moderate-intensity physical activity. This finding aligns with prior studies, which found on average that 50% of HCPs (physicians, nurses, nurse practitioners, and physician assistants) report receiving insufficient education in physical activity guidelines and prescriptions (27,28). For example, as noted in the results, more than 90% of CPA participants (94.3%) did not correctly identify coronary artery disease as the comorbidity most strongly associated with developing osteoarthritis. The finding that nearly three quarters (72.3%) of participants did not understand the *Exercise Is Medicine* 3-step approach to physical activity promotion (physical activity assessment, prescription, and referral) highlights the need for further education in this area. This finding is similar to findings in other studies that have shown gaps in primary care providers’ knowledge and implementation of physical activity counseling (29,30). Lack of confidence and training were also identified as barriers for physical activity counseling (31). Among primary care providers who discussed physical activity with their patients, only 78.7% reported assessing patients’ current physical activity levels, 25.6% wrote an exercise prescription, and 15.1% referred patients to intensive behavioral counseling (32). Furthermore, 2 in 5 primary care providers regularly assess for and recommend physical activity to their patients with arthritis (31).

The findings from the CPA identified deficiencies in knowledge of key evidence (ie, participants providing incorrect answers) for physical activity as a therapeutic intervention (54.2%–81.3%) and guideline-based care (physical activity recommendations) for people with arthritis (48.3%–75.0%). These findings show a need for strategies that support continued educational opportunities for HCPs to increase knowledge and awareness about physical activity counseling, arthritis management, and AAEBIs. When HCPs regularly assess physical activity as a vital sign, they can more easily include physical activity counseling, prescription, and referral to AAEBIs. Increasing knowledge and awareness about physical activity counseling and AAEBIs is an important strategy to increasing physical activity levels among adults with arthritis, thereby improving their health outcomes. The recommendation to participate in regular physical activity is an essential component of whole person health care for those affected by arthritis, especially given that evidence suggests that adults are more likely to attend an education program and engage in physical activity when recommended by an HCP (33,34).

### Limitations

At least 3 limitations should be considered when reviewing the results from the CPA. First, results from the CPA are representative only of those who answered the questions; participation was voluntary on a web-based continuing education platform during November 28, 2022, and December 31, 2024, and likely over-represents clinicians who are already interested in arthritis and physical activity, introducing potential self-selection and nonresponse bias. Rather than claim broad generalizability (the extent to which a study’s results can be applied to a larger, similar population), we emphasize transferability (the degree to which a reader can apply a study’s findings to their own specific context). Second, while the questions used to measure HCP knowledge have face validity, they do not fully capture all relevant constructs (eg, implementation climate, referral workflows, payer constraints), and we did not assess internal consistency, test–retest reliability, or measurement invariance; thus, it is unclear whether items carry the same meaning across specialties. Lastly, outcomes include self-reported intentions that may be influenced by factors such as local policy or procedure barriers. Future research on the impact of local policies and workflow on physical activity assessment, prescription, and referral could better distinguish educational effects from contextual constraints and strengthen claims about impact.

### Conclusions

More than 20,000 HCPs accessed the *Lifestyle Management Programs for Arthritis: Expand Your Knowledge on Evidence-Based Interventions* CPA. The diversity of learners — primary care physicians, specialty care physicians, advanced practice providers, physical therapists, nurses, and pharmacists — reflects an interest across various disciplines to address the needs of people affected by arthritis.

Especially notable was the impact of the learning intervention. Learners overwhelmingly found the faculty knowledgeable and effective, found the course free of bias, and would recommend it to a colleague. Additionally, most learners indicated a commitment to make changes in their clinical practice as a result of the knowledge attained from the learning activity. These key performance indicators demonstrate the effectiveness of the educational interventional design in supporting adult learning needs for HCPs. The CPA provided learners with the opportunity to reflect on their knowledge related to caring for people with arthritis.

The educational intervention revealed significant knowledge and competence gaps. Across all disciplines, 94.3% incorrectly answered a question related to the association of coronary artery disease as a comorbidity with the development of arthritis. Likewise, most HCPs were unfamiliar with the evidence supporting physical activity for improving pain and functionality and physical activity recommendations for patients with arthritis. The evaluation highlights the importance of ongoing education for HCPs to build awareness of AAEBIs and the assessment of physical activity as a vital sign.

The finding that HCPs need to be equipped with the necessary tools and information to counsel adults with arthritis on physical activity aligns with previous research emphasizing the need to increase knowledge and provide resources for HCPs to effectively promote physical activity and recommend evidence-based programs for self-management (29). Providing HCPs with such resources and information might increase physical activity counseling for patients with arthritis. This includes understanding the benefits of physical activity for arthritis management, being aware of evidence-based guidelines and recommendations, and having access to resources such as physical activity programs or self-management interventions specifically designed for people with arthritis. When HCPs are well-informed and equipped, they can play a vital role in encouraging adults with arthritis to engage in regular physical activity. They can provide guidance on appropriate exercises, address concerns or barriers related to arthritis symptoms, offer strategies for managing pain during physical activity, and refer patients to specialized programs or resources that cater to their specific needs. Ultimately, by empowering HCPs with the necessary tools and information, they can effectively counsel adults with arthritis on physical activity. This counseling can lead to improved patient outcomes, increased engagement in evidence-based programs, better management of arthritis symptoms, and overall enhanced quality of life for people living with this condition.

## References

[1] Fallon EA , Boring MA , Foster AL , Stowe EW , Lites TD , Odom EL , . Prevalence of diagnosed arthritis — United States, 2019–2021. *MMWR Morb Mortal Wkly Rep.* 2023;72(41):1101–1107. 10.15585/mmwr.mm7241a1 37824422 PMC10578950

[2] Hootman JM , Helmick CG , Barbour KE , Theis KA , Boring MA . Updated projected prevalence of self-reported doctor-diagnosed arthritis and arthritis-attributable activity limitation among US adults, 2015–2040. *Arthritis Rheumatol.* 2016;68(7):1582–1587. 10.1002/art.39692 27015600 PMC6059375

[3] Alizadeh S , Daneshjoo A , Zahiri A , Anvar SH , Goudini R , Hicks JP , . Resistance training induces improvements in range of motion: a systematic review and meta-analysis. *Sports Med.* 2023;53(3):707–722. 10.1007/s40279-022-01804-x 36622555 PMC9935664

[4] Murphy JR , Di Santo MC , Alkanani T , Behm DG . Aerobic activity before and following short-duration static stretching improves range of motion and performance vs. a traditional warm-up. *Appl Physiol Nutr Metab.* 2010;35(5):679–690. 10.1139/H10-062 20962924

[5] Verlaan L , Bolink SA , Van Laarhoven SN , Lipperts M , Heyligers IC , Grimm B , . Accelerometer-based physical activity monitoring in patients with knee osteoarthritis: objective and ambulatory assessment of actual physical activity during daily life circumstances. *Open Biomed Eng J.* 2015;9(1):157–163. 10.2174/1874120701509010157 26312077 PMC4541405

[6] Centers for Disease Control and Prevention. Chronic disease indicators, activity limitation due to arthritis among adults aged >=18 years who have doctor-diagnosed arthritis. Behavioral Risk Factor Surveillance System (BRFSS). 2021. Accessed December 1, 2023. https://nccd.cdc.gov/cdi/rdPage.aspx?rdReport=DPH_CDI.ExploreByTopic&islTopic=ART&islYear=9999&go=GO

[7] Smith TO , Higson E , Pearson M , Mansfield M . Is there an increased risk of falls and fractures in people with early diagnosed hip and knee osteoarthritis? Data from the Osteoarthritis Initiative. *Int J Rheum Dis.* 2018;21(6):1193–1201. 10.1111/1756-185X.12871 27153388

[8] Murphy LB , Cisternas MG , Pasta DJ , Helmick CG , Yelin EH . Medical expenditures and earnings losses among US adults with arthritis in 2013. *Arthritis Care Res (Hoboken).* 2018;70(6):869–876. 10.1002/acr.23425 28950426

[9] US Department of Health and Human Services. Physical activity guidelines for Americans, 2nd edition. Accessed January 27, 2026. https://odphp.health.gov/sites/default/files/2019-09/Physical_Activity_Guidelines_2nd_edition.pdf

[10] Ambrose KR , Golightly YM . Physical exercise as non-pharmacological treatment of chronic pain: why and when. *Best Pract Res Clin Rheumatol.* 2015;29(1):120–130. 10.1016/j.berh.2015.04.022 26267006 PMC4534717

[11] Dasso NA . How is exercise different from physical activity? A concept analysis. *Nurs Forum.* 2019;54(1):45–52. 10.1111/nuf.12296 30332516

[12] England BR , Smith BJ , Baker NA , Barton JL , Oatis CA , Guyatt G , . 2022 American College of Rheumatology guideline for exercise, rehabilitation, diet, and additional integrative interventions for rheumatoid arthritis. *Arthritis Care Res (Hoboken).* 2023;75(8):1603–1615. 10.1002/acr.25117 37227116

[13] Osteoarthritis Action Alliance. Physical activity AAEBI cross-sectional table. Accessed October 17, 2025. https://oaaction.unc.edu/wp-content/uploads/sites/623/2023/10/Physical-Activity-AAEBI-Cross-Sectional-Chart-2023.pdf

[14] Lucero KS , Williams B , Moore DEJ Jr . The emerging role of reinforcement in the clinician’s path from continuing education to practice. *J Contin Educ Health Prof.* 2023;44(2):143–146. 10.1097/CEH.0000000000000541 37962911 PMC11107885

[15] Centers for Disease Control and Prevention. Physical activity and self-management education programs for arthritis. Accessed January 17, 2026. https://www.cdc.gov/arthritis/programs/index.html

[16] Feehan L , Westby M. Patients with rheumatoid arthritis should exercise more, sit less. *The Rheumatologist.* 2014;Nov.

[17] Osteoarthritis Action Alliance (OAAA), Thurston Arthritis Research Center (TARC). Arthritis-appropriate, evidence-based interventions (AAEBI). Osteoarthritis Action Alliance. Accessed December 1, 2023. https://oaaction.unc.edu/aaebi/

[18] Fallon EA , Brown DR , Callahan LF , Foster AL , Kim JS , Lo GH , . Stepping up counseling and referral to effective physical activity interventions for adults with osteoarthritis. *J Rheumatol.* 2024;51(2):209–212. 10.3899/jrheum.2023-0693 37967909 PMC11740016

[19] Brady TJ , Jernick SL , Hootman JM , Sniezek JE . Public health interventions for arthritis: expanding the toolbox of evidence-based interventions. *J Womens Health (Larchmt).* 2009;18(12):1905–1917. 10.1089/jwh.2009.1571 20044851

[20] Dunn AL , Marcus BH , Kampert JB , Garcia ME , Kohl HW III , Blair SN . Comparison of lifestyle and structured interventions to increase physical activity and cardiorespiratory fitness: a randomized trial. *JAMA.* 1999;281(4):327–334. 10.1001/jama.281.4.327 9929085

[21] Elgaddal N , Kramarow EA , Reuben C . Physical activity among adults aged 18 and over: United States, 2020. *NCHS Data Brief.* 2022;(443):1–8. 36043905

[22] Graham RC , Dugdill L , Cable NT . Health professionals’ perspectives in exercise referral: implications for the referral process. *Ergonomics.* 2005;48(11-14):1411–1422. 10.1080/00140130500101064 16338709

[23] Dunphy R , Blane DN . Understanding exercise referrals in primary care: a qualitative study of general practitioners and physiotherapists. *Physiotherapy.* 2024;124:1–8. 10.1016/j.physio.2024.04.348 38776568

[24] Medscape. Who we are. Accessed January 5, 2024. https://www.medscape.com/public/about

[25] Verga Răuță GI , Baltă AAȘ , Ciortea D-A , Petrea Cliveți CL , Șerban Grădinaru M , Matei MN , . Healthcare interventions in the management of rheumatic diseases: a narrative analysis of effectiveness and emerging strategies. *Healthcare (Basel).* 2025;13(14):1691. 10.3390/healthcare13141691 40724716 PMC12294379

[26] Health care providers’ action guide. American College of Sports Medicine, Exercise Is Medicine. Accessed January 17, 2026. https://www.exerciseismedicine.org/wp-content/uploads/2021/02/EIM-Health-Care-Providers-Action-Guide-clickable-links.pdf

[27] Grimstvedt ME , Der Ananian C , Keller C , Woolf K , Sebren A , Ainsworth B . Nurse practitioner and physician assistant physical activity counseling knowledge, confidence and practices. *Prev Med.* 2012;54(5):306–308. 10.1016/j.ypmed.2012.02.003 22349645

[28] Douglas F , Torrance N , van Teijlingen E , Meloni S , Kerr A . Primary care staff’s views and experiences related to routinely advising patients about physical activity. A questionnaire survey. *BMC Public Health.* 2006;6:138. 10.1186/1471-2458-6-138 16719900 PMC1523207

[29] Shuval K , Leonard T , Drope J , Katz DL , Patel AV , Maitin-Shepard M , . Physical activity counseling in primary care: insights from public health and behavioral economics. *CA Cancer J Clin.* 2017;67(3):233–244. 10.3322/caac.21394 28198998

[30] Wattanapisit A , Wattanapisit S , Wongsiri S . Overview of physical activity counseling in primary care. *Korean J Fam Med.* 2021;42(4):260–268. 10.4082/kjfm.19.0113 32429011 PMC8321902

[31] Guglielmo D , Murphy LB , Theis KA , Helmick CG , Omura JD , Odom EL , . Physical activity assessment and recommendation for adults with arthritis by primary care providers — DocStyles, 2018. *Am J Health Promot.* 2021;35(4):559–570. 10.1177/0890117120981371 33356415 PMC10479947

[32] Omura JD , Bellissimo MP , Watson KB , Loustalot F , Fulton JE , Carlson SA . Primary care providers’ physical activity counseling and referral practices and barriers for cardiovascular disease prevention. *Prev Med.* 2018;108:115–122. 10.1016/j.ypmed.2017.12.030 29288783 PMC5870116

[33] Murphy LB , Theis KA , Brady TJ , Sacks JJ . Supporting self-management education for arthritis: evidence from the Arthritis Conditions and Health Effects Survey on the influential role of health care providers. *Chronic Illn.* 2021;17(3):217–231. 10.1177/1742395319869431 31475576 PMC10878350

[34] Duca LM , Helmick CG , Barbour KE , Guglielmo D , Murphy LB , Boring MA , . Self-management education class attendance and health care provider counseling for physical activity among adults with arthritis — United States, 2019. *MMWR Morb Mortal Wkly Rep.* 2021;70(42):1466–1471. 10.15585/mmwr.mm7042a2 34673750 PMC9361837

